# Bird nest building: visions for the future

**DOI:** 10.1098/rstb.2022.0157

**Published:** 2023-08-28

**Authors:** Susan D. Healy, Maria Cristina Tello-Ramos, Marie Hébert

**Affiliations:** School of Biology, University of St Andrews, Harold Mitchell Building, St Andrews, Fife KY16 9TH, UK

**Keywords:** brain, building, development, evolution, hormones, nest

## Abstract

Successful reproduction for most birds requires them to have built ‘good’ nests. The remarkable diversity of nests across approximately 10 000 species of living birds suggests that ‘good’ nest design depends critically on a species' microhabitat, life history and behaviour. Unravelling the key drivers of nest diversity remains a key research priority—bolstered by renewed appreciation for nest museum collections and increasing correlational field and experimental laboratory data. Phylogenetic analyses—coupled with powerful datasets of nest traits—are increasingly shedding light on the evolution of nest morphology and there are functional questions yet to be addressed. For birds, at least, developmental and mechanistic analyses of *building* (behaviour, hormones, neuroscience) itself, rather than measurements and analyses of nest *morphology,* are already becoming the next major challenge. We are moving towards a holistic picture in which Tinbergen's four levels of explanation: evolution, function, development, and mechanism, are being used to explain variation and convergence in nest design—and, in turn, could shed light on the question of how birds know how to build ‘good’ nests.

This article is part of the theme issue ‘The evolutionary ecology of nests: a cross-taxon approach’.

## 1. Introduction

As is evident from the diversity of papers in this issue of *Philosophical Transactions B,* nests are built by a wide variety of animals, and for diverse purposes. However, probably the best-known group for their nests, not least for their ubiquity and conspicuousness, is birds. So it is on birds that we focus here. But we consciously take a different approach to nests than is typical: here we address the process by which nests come about—nest building. For all the interest in nests over the past decades, there has been, surprisingly to us, at least, very little interest in building (other than for fish e.g. [1]). We propose that the 'four questions framework' provided by Tinbergen (evolution, function, development and mechanism), coupled with a diversity of methods enabling unprecedented access to building, promises a question-rich future for our understanding of nests, and not just in birds.

In 1867, Alfred Russel Wallace wrote that he did not believe that birds built their nests by instinct [2]. Almost 40 years later, Charles Dixon wrote a book introducing the science of caliology, or the study of birds' nests [3]. In it, he provided a wealth of information about bird nests: locations they could be found, materials used, the morphology of the final object. He also described multiple instances that demonstrated, incontrovertibly in his view, that instinct was no more likely to explain bird nest building than it explains building by humans. Both Dixon and Wallace made direct comparisons of avian nest building with examples of human building and neither could find evidence that the mechanisms (then labelled 'intelligence' rather than the now more common label, 'cognition') that enable birds to build were significantly different from those enabling humans to build.

Some 150 years later, however, the prevailing views of nest building are diametrically opposed to these of Wallace and Dixon. Indeed, the view is that nest building in most, if not all, animals is an instinctive behaviour (e.g. see this and other 2023 websites: https://journeynorth.org/tm/robin/BuildNest.html). Wallace and Dixon might both be at least a little surprised. In fact, until this (the 21st) century, there has been really rather little interest directed at what birds might ‘know’ about nests and how to build them, with a small handful of experiments, field observations and comparative analyses dotted over the later part of the past century. That instinct has become such a prevalent explanation may have come about for two reasons. The first comprises work carried out around the 1950s–1970s in which individuals of a range of bird species were raised by hand [4]. Hand-raising allows manipulation of early experience and several researchers looked to see what kinds of nest the birds they had hand-raised went on to build when sexually mature. What the birds produced varied, depending on the species. However, only the work by Collias & Collias provided much in the way of quantitative data [5,6]. They found that hand-raised village weavers *Ploceus velatus* built nests that looked rather like a typical village weaver nest, although those birds also went on to build much neater nests as they built more nests. This suggested a role for experience.

However, ever since Wallace and Dixon there has been considerably more interest in studying the physical structure, the nest itself, rather than in the behaviour that produces it, the latest evidence of which is provided in nearly all of the papers in this issue. One of the rare exceptions is the work conducted on nest building in fish (e.g. [7]; [8] in this issue; [9,10]) and the more recent work on songbirds (e.g. [11–15]). Studying the behaviour that produces nests rather than the artefact itself is definitely a focus for the present and for the future, not least because of the logistic issues that surround both the collection and analysis of building data. Of which more later.

Does it matter that ‘nest building’ is quite commonly used to describe morphological rather than behavioural data, even when those data are of nest morphology (e.g. [16–19])? Interest in avian nest morphology rather than behaviour makes a lot of sense: bird nests are often regarded as things of beauty, they have been raided for the eggs they contain for centuries, and they are much easier to measure, not least because they have been kept in multiple museum collections. They also have the considerable advantage over behaviour for their durability and longevity: they are designed to last long after the behaviour creating them has finished, and they can last for years in use, or over decades in storage. These features allow their function to be tested *in situ* or in a laboratory, their material makeup and their morphology to be identified and measured, and for comparisons to be made. Bird nests (along with beaver dams) were also one of the prominent examples of an extended phenotype discussed by Richard Dawkins in his book of the same name [20]. Dawkins' proposal was that animals can modify their environment beyond their phenotype (thus 'extended') via their architectural constructions. And indeed, data from inside bird nests show that the builders can, for example, warm the nest and its contents well above the ambient temperature [21,22]. Forty years and a bit from that publication, it seems time enough to include here questions as to whether considering nests as extended phenotypes has advanced or is advancing our understanding of their evolution. And what more might be made of both evolutionary analyses and of examination of nest function?

## 2. The evolution of nest building via comparative analyses

Prior to the notion of extended phenotypes, another Oxford luminary, Niko Tinbergen, had already outlined a framework for examining animal behaviour that continues to shape the form and direction of the field of animal behaviour (e.g. [23–26]). Because of the attributes of nests rather than of building itself, of Tinbergen's four questions, it has been the evolutionary and functional questions that have received most attention from nest researchers. The application of comparative methods to questions concerning variation in interspecific nest morphology has shown that a variety of factors have probably shaped nests (quite literally) from the morphology of the builders (e.g. beak shape in birds: [27]), to some of the builders' behaviours (e.g. collective foraging in ants: [28]). The value of such comparative analyses can be much more fully realised if they are used to direct empirical data collection. For example, that both bird beaks and ant foraging are correlated with the morphology of the artefacts made by both groups brings strength to a recent suggestion by Sugasawa *et al*. [29,30] regarding our understanding of object manipulation and dexterity. Currently that understanding largely depends on how primate hands work, but bird beaks and ant mandibles seem capable of manipulating diverse objects into functional structures, structures that rival objects made by human hands. Perhaps robotics might look to nest building for new design ideas for gripping, twisting, even weaving?

In order to achieve this, however, we need detailed interspecific quantification of object manipulation in addition to the increasing number of studies that have used large phylogenetic datasets on nest morphology. One key advantage of these large comparative analyses is that they allow an estimate of the speed at which the morphology of nests changes across evolutionary time, and the direction in which the morphology tends to go. Probably because they have been conducted on different datasets, this has led, for example, to variation in descriptions of the evolution of cup and domed nests and the causes of the change from one to another. While it appears that domed nests are ancestral to cup nests in passerines [31], both Fang *et al*. [32] and Ocampo *et al*. ([33], this issue) provide evidence that suggests multiple examples of ‘reverse evolution’ in which domes have evolved from cup nests. Why domed nests might evolved, in the Old World babblers (the Timaliidae) at least, seems to depend on whether birds build their nests on or near the ground, where it seems plausible that domed nests are useful as a means to reduce predation. Across time for this group it appears that domed nests built in trees either become open nests or birds move to the ground (perhaps building domed nests where they are not especially useful is too costly?) while cup nest design moves speedily in the opposite direction [34]. In the clade Tyrannidae, on the other hand, habitat (e.g. closed or open) does not corelate with the presence or absence of domes on nests [33]. Also recently, Fang *et al*. [32] showed how nest structure changes relative to the location and attachment of nests, all of which appear to change independently of each other.

One feature common to comparative studies is that the sample size (number of species) can be very dependent on the question being addressed. If the questions are reasonably straightforwardly asking about how morphology changes, then the available data may incorporate almost all 249 families of birds (using birds as an example). If, however, one attempts to ask what might have caused one or other morphology to evolve or to vary (as asked by [35] in this issue), then sample size begins to diminish quite quickly. For example, Perez *et al*. [35] began with 55 species, but some analyses contained only 35 species, while for Vanadzina *et al*. [36] some analyses contained as many as 965 species, but others contained only 374. These studies have only begun to scratch at the surface of the evolution of nest morphology and as pointed out by Fierro-Calderon *et al*. [37], even for nest morphology, there are many species for which we are yet to have a species description.

## 3. The value of museum specimens

Although this is not so feasible for fish and for groups of animals that lay eggs in scrapes, or holes, or dig burrows, there are many species for which their nests can be removed from the habitat intact and taken into museum collections. Of course, museum egg collections have long proved a rich and invaluable source of data for a range of questions on reproduction, particularly of birds, but the vessel in which those eggs were contained features to a much lesser extent, both in the collections and in the literature [38]. Nonetheless, although slow to get underway, there have been an increasing number of studies utilizing birds' nests that are held in various museums. The Natural History Museum in Tring, UK, has several thousand nests—and for some species, multiple nests—enabling assessments of intraspecific variation in nest morphology (e.g. [35], this issue). The number and species coverage of nests in this collection alone enable much more substantial comparative analyses than has been typical (see [36]).

Museum nest collections also possibly enable assessments of changes in the environment, not just in the nest itself. For example, Australian museum nests have provided data on the increase in plastic prevalence across a century and a half (an increase from 4% in 1832 to nearly 30% in 2018: [39]). It remains to be seen whether this considerable increase in plastic content in nests is always detrimental. Although entanglement of young in plastics is recorded in seabirds [40,41], it is possible that plastics and other anthropogenic materials are either not costly once included in the nest, or even beneficial if their insulative capacities or their mechanical properties actually lead to a nest that is warmer/stronger than one that contains no anthropogenic materials [42,43]. While geographic comparisons of the inclusion of such materials may already be increasing [44], museum collections might usefully add a temporal component to any evidence of change. Potvin's [45] analysis of 250 museum nests, which showed that increasing mud content reduced the noise levels detectable inside the nest, suggests that other ingenious uses of museum nests might address questions of the impacts of anthropogenic modifications to the environment, both across time and space.

Conversations about nests are often peppered with ‘we don't know the answer to that’, so perhaps it should not be surprising that despite the very many studies on eggs and their various attributes, often incorporating museum specimens, there have, thus far, been very few broadscale studies of the relationship between egg colour and nest characteristics, and the recent ones have all suggested complex relationships between egg coloration and nest morphology [46–48]. There are not yet any clear answers, but the museum collections of nests and eggs are going to come in handy for further exploration of this relationship.

Finally, new methodologies are being developed or applied to nests in museum collections enabling an increasing diversity of questions to be addressed. These methods include combining deconstruction of nests together with photographic analysis to assess whether nest photographs would be useful for identifying and quantifying materials contained within them [29]. This could mean that photographs of nests taken in the wild could provide useful quantitative data on nest materials in addition to, or even in place of, nest removal and deconstruction. Sequencing methods applied to nest materials is allowing the identification and comparison of contemporary and historical plants associated with the location/habitat in which the nest was found. Rinkert *et al*. [49] have suggested this information could be useful for environmental restoration plans.

## 4. Nest function

**Figure 1.**
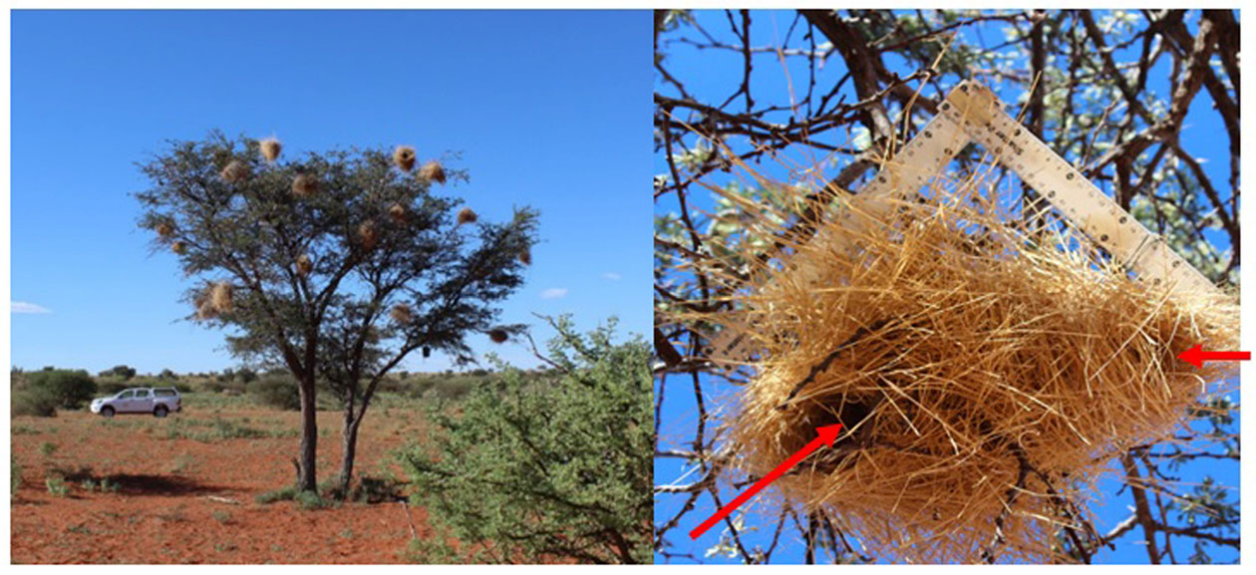
Left panel: a tree in the Kalahari Desert, South Africa in which multiple roosts have been built by a group of white-browed sparrow weavers (*Plocepasser mahali*). Right panel: close-up of a white-browed sparrow weaver roost. The red arrows point to the entrance (arrow on the right) and the exit (arrow on the left). (Photographs by Maria Cristina Tello-Ramos.)

It might be assumed that a section on the function of nests would be pretty short: this is one question for which we should have the answer already, right? Bird nests (and those of most other animals) are for protecting the eggs, growing chicks and parents from predation and/or the abiotic environment, for sexual display, or for practising building [50]. At least some birds do more than protect their young from the abiotic environment when the nest is used to increase the temperature at which eggs are incubated to one that enables the most effective incubation (e.g. [51,52]). Some structures that closely resemble nests may actually just be used for roosting, as are the multiple roosts built by white-browed sparrow weavers *Plocepasser mahali* (figure 1). And this list is probably pretty much it. So, yes, this is going to be a short section.

But there is a considerable future to be had in this area as there is still much to be learned about the functionality of the materials used (as already mentioned with respect to anthropogenic materials), the amount of material included, the value of a dome over a cup nest, the value of building a nest inside a hole, and more [53]. There are also very few data that demonstrate the adaptive value of these nest features as to do so requires putting quantitative data on materials included together with the consequent reproductive success of the builder (e.g. [54]), as well as the relationship between the effort a builder puts into building relative to the effort that may subsequently be expended in incubation of the eggs. For some species, the effort is all in the building [55] but for others building is only part of their reproductive expenditure. For birds, at least, we know very little about the energetic expenditure involved in all of the activities that result in the production of a nest, other than that we assume that building is a costly business. There is a lot of work to be done in these various regards. Experimental manipulations such as removing materials or parts of nests will also be useful to determine whether, for example, avian builders ‘over-engineer’ their nests—perhaps not with insulation as they do not want to cook their young, but maybe it would be sensible for the builders to be absolutely sure about structural materials or attachments. No parent wants a nest to fall off/down, for its bottom to fall out or for it to float/blow away: predators and the weather are not actually the only problem that a builder needs to solve.

## 5. Development of nest building

One building problem that we thought we had solved is really just one that we have largely ignored. That is the role of development, and how a builder becomes proficient in the choice of appropriate location and materials, and in the act of building itself. We might also ask how a naïve builder knows what to build. Although song is now known to be largely a learned behaviour, we know very little about what young birds learn about building. Anecdotal reports of juvenile male weavers manipulating material in nests being built by their dad or other adult males might suggest that these young males attend to building (while young females do not?) and may be practising something. The few available data show that dexterity in handling materials does increase with material manipulation or ‘mandibulation’ (all data on weavers: [5,6,56,57]). Whether, and if so how, early-life experience of materials typically affect nest-building proficiency across species remains to be seen, including in non-avian species.

That there is, or can be, a social component to nest-building decision-making is currently based on observational data and a small handful of experimental studies both from the laboratory and the field. Location choice, based on predation avoidance [58] or on territory quality (e.g. [59,60]), appears to be a nest-building decision that can be made on the basis of information gathered from either conspecifics or heterospecifics. However, that this does not always occur shows that data from more species and locations are needed before generalizations can be made as to the use of such information [61].

Social learning about building can occur both prior to sexual maturity and when building a first nest: exposure to materials prior to sexual maturity affects the rate of nest building when the birds later build their first nest, but only when the materials were provided along with an (unrelated) adult male (zebra finches *Taeniopygia guttata*: [62]). In addition, naive sexually mature avian nest builders copy the material choices of experienced birds, although, at least in zebra finches, only if the builders are familiar to them [14,63]. How widespread is the copying of any aspect of building is a question for future exploration; these explorations might include consideration of whether the species concerned is social or has the opportunity to observe building by others (e.g. in a PIT-Tagged mixed-species community of tits (Paridae sp.), birds foraged and collected nest material together: [64]) or they might just observe nests being built by others, particularly conspecifics [65].

## 6. The mechanistic basis of nest building, especially hormones and the brain

### (a) Endocrinology

Increasing builder proficiency through practice or observing the skills of others might lead to the building of a good nest, but how does the builder know when their nest is good enough? If the builder is male and is building for a female, what does she use to decide that he has built her an excellent/good/good enough nest?

The contexts in which cognition plays a role in animal behaviour have steadily been increasingly recognized. And nest building is no exception. As learning and its possible roles in nest building are considered by Lehtonen *et al*. in this issue [66], here we turn to two different but also key mechanisms underpinning building: hormones and neurobiology.

The hormonal control of reproductive behaviours in birds, investigated in detail since the 1960s, enables an integrated cascade of events in which hormones and behaviours influence one another in turn, in both the target individual and its partner. Nestbuilding is an especially interesting stage of reproduction because it sits between the courtship/territory/mate defence stage, which is typically characterized by high circulating testosterone levels in males and aggression directed at other males, and the parental stages, which may include incubation as well as brooding and feeding the chicks (at least in altricial species). In some species, this transition stage may be rather short, at least in comparison with the stages of territory establishment/courtship and offspring care. And yet, again, extraordinarily little is known about the hormonal changes that occur during this important transition period, and how these changes interact with a builder's behaviour or that of its mate. There are some data from experiments involving castration in male ring doves (*Streptopelia risoria*) that show that testosterone, applied either to the peripheral circulation or to a specific part of the hypothalamus (the preoptic area), restores the castration-induced loss of nest building in this species [67,68]. What is more, peripheral administration of oestradiol to the males has the same effect, which is interesting as the activity of the enzyme responsible for the conversion of testosterone into oestradiol (i.e. aromatase) is known to drastically increase in this hypothalamic area following courtship [69]. In overactomized females, the co-administration of both oestradiol (whose production is usually elevated during the courtship period; [70]) and progesterone in the circulation seems needed to restore nest building, which in turn facilitate males' building behaviour [71].

By its very nature, this kind of work is much more readily undertaken in the laboratory with species like ring doves that will breed in captivity, so it is perhaps not surprising that zebra finches and canaries (*Serinus canaria*) contribute most of the rest of our knowledge of the neuroendocrinology of nest building. While those data seem mostly to corroborate the observations made in ring doves, they also show a degree of species-specificity. For example, as found in doves, the presence of the male influences both a female canary's reproductive physiology and her behaviour: a male, or at least the acoustic cues he provides, seems to facilitate both female nestbuilding and the development of her reproductive tract [72,73]. Unlike ring doves however, oestradiol alone seems to be sufficient to support nest building by female canaries, and although males usually contribute little to building in this species, oestradiol administered to castrated male canaries appears to prompt them to build a nest that is indiscernible from that built by females [74]. In zebra finches, although male courtship behaviours can be fully restored by the administration of aromatizable androgens, (i.e. androgen such as testosterone that can be converted into oestrogens by the enzyme aromatase), both androgenic and oestrogenic metabolites seem to be co-involved as administration of either non-aromatisable androgens or oestrogens alone have little effect [75]. Factors that reduce circulating levels of testosterone in males, such as water restriction (water availability is a trigger for breeding in this opportunistic breeder; [76]), also reduce the occurrence of nestbuilding [77]. These effects are consistent with the reported role of aromatisable androgens (e.g. testosterone) in rescuing/promoting nestbuilding [78] and nest-oriented behaviours [79] in males (the main building sex in this species).

A general conclusion that can be drawn is that sex-steroid hormones are tightly associated with early breeding events, including nestbuilding. Among them, oestradiol always seems to prompt nestbuilding, irrespective of who is/are the builder(s) in the pair, even if it sometimes needs to be associated with other hormones. But thus far, the (neuro)endocrine mechanisms promoting nestbuilding in birds have been investigated in a handful of laboratory-raised/domesticated species, because their (i) physiological states can be easily altered; (ii) behaviour can be relatively easily monitored, and in a consistent and reproducible way; (iii) hormonal profiles according to breeding stages are relatively well characterized. Although these ‘model species’ have proved invaluable for gaining a better understanding of the hormonal actors supporting nest building, birds are characterized by a broad spectrum of breeding and nest-building strategies: nests can be built primarily by one or the other member of a pair while the other might or might not provide assistance (e.g. females: canaries and males: zebra finches), exclusively by one of the two sexes (e.g. female: blue tits *Parus caeruleus*, male: ostriches *Struthio camelus*), or duties can be shared more equally either between pair members (with tasks being split being the two sexes e.g. ring doves, or with both sexes contributing equally to all aspects of nest building e.g. jackdaws *Corvus monedula*), or even sometimes between members of a group (e.g. white-browed sparrow weavers *Plocepasser mahali*, a cooperatively breeding species in which both the breeding pair and their helpers participate in building). Collection of both behavioural and hormonal data from a broad range of non-model species would allow identification of commonalities and differences in terms of hormonal correlates of nest building, and account for both species and sex specificities. While fundamental questions could thus be addressed, we might also consider more applied questions such as the ways in which domestication affects sex-steroid production (domesticated zebra finches: [80]) and, thereby, nestbuilding in domesticated species.

### (b) The brain

The bird brain has delivered some of the most resounding impacts on our understanding of brain structure and function. For example, sex differences in brain structures were first most clearly demonstrated in bird brains in association with song learning [81]. This work has led to iconic examples of the relationship between experience and both cell birth (neurogenesis) and programmed cell death (apoptosis) and continues to shine light on how brains and behaviours are integrated [82]. Investigating brain structure and function in association with nest building may prove to be the next major contribution to come from the bird brain. This is because of the interest in physical cognition, the demonstration that cognition is involved in building, and the structural diversity of nests.

The discovery of tool making by New Caledonian crows (*Corvus moneduloides*) in the mid 1990s suddenly escalated the interest in physical cognition in animals [83] as no longer being a human, or even a primate-centric, set of behaviours but something even a bird could do (we will ignore spitting in archerfish, web-building in spiders and so on). Being a 'bird brain' stopped being an epithet. If a bird could make tools, a proper re-think of brains, cognition and behaviour is required. However, for those interested in the evolution of tool making, tool making is frustratingly uncommon, albeit taxonomically quite widespread. And here nestbuilding becomes of interest because this behaviour is a phenotype that bears considerable similarities to tool-making/tool use. Add the increasing evidence that cognition plays a not inconsiderable role in nestbuilding, and here is physical cognition occurring under our noses, at least every spring, in every habitat. We can even make it happen in laboratory species like zebra finches, and hey presto, gain access to understanding where activity occurs in the brain during building, such as the identification of relevant motor circuits as in the anterior motor pathway activation seen in nest-building zebra finches [84]. We might even see a role for whole brain regions like the cerebellum, which is increasingly implicated not only in fine motor control but also in some aspects of learning and memory [85]. Each of these levels of analysis is feasible during observations of building, during observations of nests, and other experimental manipulations of building.

While we may not be quite yet able to access the neural activity underpinning the broad variation in building, both the behaviours of building (from scraping materials together by brush turkeys (*Alectura lathami*), through head shaking while applying mud by nest-building swallows (Family Hirundinidae), to weaving with bills and feet by Southern masked weavers (*Ploceus velatus*)) through to the production of wildly various structures (the scrapes of ostriches, through the feathered/spider-webbed cups of hummingbirds to the apartments of sociable weavers), data from model systems such as zebra finches will help us direct our search more productively. Then we can ask questions such as whether more cognitive effort is required to weave a ‘complex’ nest of a weaverbird than to build the nest cup of a blackbird or the mound of a brush turkey. Or whether this structural complexity is a result of some species having better fine motor control, better coordination of their bill and feet, or more useful placement of their eyes.

## 7. Conclusion

The future for nests and nest-building research seems bright. There are lots of questions to be addressed, and they come in such a diversity that there is something for everyone, be their interests evolutionary, functional, developmental, or mechanistic. There is a diversity of species and geographical locations, and with increasing technology enabling access to behaviour in the field, so many more of these species and locations are becoming within reach. There are, of course, difficulties, with perhaps the biggest in the very relationship between nestbuilding and the nest. Which is the pertinent phenotype? Selection may act most obviously on the nest structure and it is therefore on the nest that almost all of the attention has been focussed. Not so with our view to the future: here we have deliberately focussed on building. Although most research into building has been done on the nests built by birds, this special issue highlights the growing interest in understanding the diversity of nest forms and the behaviour that produces them across taxa. Fischer, for example, invites us to include nesting behaviour of anurans to understand broader evolutionary questions about nesting behaviour given the unique life cycle of frogs and toads [86]. A life cycle that moves between wet and dry environments means that the nests they build might be subject to selective pressures common to many different taxa. As with the study of birds' nests however, little is known about the actual building behaviour of different species. Similarly, Lehtonen *et al*., in this issue [66], discuss the comparative potential of studying the cognitive abilities that enable building across animals. While there is no doubt that some elements of building might be innate, it is now clear that individual and social experiences change the nests different species build, from bees, fish and turtles to rats. We argue that the first step towards understanding the role that cognition plays in building is to describe the behaviour. Building we understand so little, but it will only be by understanding the behaviour, how and what a bird knows what to build, that we will be able to relate the nest to the builder's genotype. For that problem to be unpicked, there needs to be a much bigger focus on building. This will require more time spent collecting field observations, more experimental manipulations—many of which will need to be in the laboratory (at least at the beginning)—and probably an awful lot of video analysis. We suspect that included among the variety of features that these data will reveal will be a considerable level of intraspecific variation in building and in the resulting nest, much more evidence of experience-dependence, but also importantly more data for comparative analyses. And if all of this is not sufficient incentive for working on nests and nest building, then we finish with asking how it is in 2023 that we know the identity of the builder in only some 20% of bird species?

## Data Availability

This article has no additional data.
